# Growing networks with communities: A distributive link model

**DOI:** 10.1063/5.0007422

**Published:** 2020-04-23

**Authors:** Ke-ke Shang, Bin Yang, Jack Murdoch Moore, Qian Ji, Michael Small

**Affiliations:** 1 Computational Communication Collaboratory, Nanjing University, Nanjing 210093, People’s Republic of China; 2 College of Information and Communication Engineering, Dalian Minzu University, Dalian 116600, People’s Republic of China; 3 School of Physical Science and Engineering, Tongji University, Shanghai 200092, People’s Republic of China; 4 Complex Systems Group, Department of Mathematics and Statistics, The University of Western Australia, Crawley, Western Australia 6009, Australia; 5 Mineral Resources, CSIRO, Kensington, Western Australia 6151, Australia

## Abstract

Evolution and popularity are two keys of the Barabasi–Albert model, which generates a power law distribution of network degrees. Evolving network generation models are important as they offer an explanation of both how and why complex networks (and scale-free networks, in particular) are ubiquitous. We adopt the evolution principle and then propose a very simple and intuitive new model for network growth, which naturally evolves modular networks with multiple communities. The number and size of the communities evolve over time and are primarily subjected to a single free parameter. Surprisingly, under some circumstances, our framework can construct a tree-like network with clear community structures—branches and leaves of a tree. Results also show that new communities will absorb a link resource to weaken the degree growth of hub nodes. Our models have a common explanation for the community of regular and tree-like networks and also breaks the tyranny of the early adopter; unlike the standard popularity principle, newer nodes and communities will come to dominance over time. Importantly, our model can fit well with the construction of the *SARS-Cov-2* haplotype evolutionary network.

Evolution and popularity are two keys of the Barabasi–Albert (BA) model—the default model for generation and growth of complex networks, with a power law distribution of network degrees. Evolving network generation models are important as they offer an explanation of both how and why complex networks (and scale-free networks, in particular) are ubiquitous. However, the motivation and manifestation of “popularity,” the other key to the BA model, are often rather ambiguous for natural networks. We also argue that the “rich-get-richer” principle is not necessarily sufficient or ubiquitous across all phenomena in all biological, social, and physical networks or particularly in engineering networks. Moreover, the growth of mesoscale network structures—communities—remains not fully understood. Here, we develop a growing network model with new communities for regulating the degree distribution, and new communities will absorb link resources to weaken the degree growth of hub nodes. Interestingly, our framework can construct a tree-like network with clear community structures—the branches and leaves of a tree. Results show that our models have a common explanation for the community of regular and tree-like networks and can also regulate degree distributions via a free parameter. We show that our model is also capable of replicating the features of real biological systems—it can construct a network that has a similar structure to the SARS-Cov-2 haplotype evolutionary network.

## I. INTRODUCTION

Evolving networks are ubiquitous,^1–6^ from social networks^7^ to protein networks,^8^ from the Internet^9^ to contact networks,^10^ and so on. Hence, in all these instances, time is the basic element for a fitness network model.^11,12^ However, another basic element, the principle of preferential attachment—leading to the scale-free degree distribution—is increasingly controversial^13,14^ as an omnipresent model of complex systems. In one recent study, it was shown that the scale-free network is rare.^13^ In this report, we argue that time evolution is the key for any network model, but the birth time of a node should not entirely preordain its final state. While the first mover advantage is an advantage, it is not sufficient to guarantee final success—sometimes better ideas will appear later. In particular, the degree distribution of growing engineering networks is usually influenced by both geography and resource-driven demand; hence, links cannot be predicted well via an algorithm that is based solely on the principle of pure preferential attachment.^15,16^

On the other hand, communities appear in many complex networks and are frequently associated with important functions of those networks.^17–20^ Research into community detection also has recently attracted increasing attention.^21,22^ Actually, the appearance of new communities will pillage the degree resource from old hub nodes, as the links within communities are denser than those between groups,^17^ and a new hub node is easily formed with the new community.

In this report, we adopt two keys—network evolution and new communities—to design a growing network model. With time evolution, upstarts (new nodes with high degrees) will appear in new groups, and our model provides a novel perspective on the “rich-get-richer” phenomenon. First, we construct a fully connected network, and every node is one initial community. Second, we evaluate the probability that generates a new community or choose a community by the same probability mass function. Third, we introduce a new node that connects nodes within the new or chosen community with a higher probability and external nodes of that with a lower probability. Fourth and finally, we repeat steps 3 and 4 as required.

In what follows, we show network structures that are constructed via our model and their degree distributions. In regular networks (networks with many crossing links^16^), with the increasing probability of the appearance of new communities, the degree of the hub node decreases—consistent with the hypothesis of our model. Surprisingly, our model can also construct a tree-like network with natural increasing new branches (communities).

To investigate the community structure of our model more carefully, we measure the visibility of community structures by modularity.^23^ We find that networks that are constructed via our model with appropriate parameters have obvious community structures. Furthermore, we detect the predictability of communities and find that links within a community are more predictable than those between communities. All in all, our model can construct a regular or tree-like network with clear and predictable communities and controllable degree distributions. In the end, we successfully construct a tree-like network with a similar structure to the *SARS-Cov-2*^24^ haplotype evolutionary network and a regular network with a similar structure to the karate club network.

## II. GROWING MODEL

(1) Given k(k≥1) nodes, construct a fully connected network, where each node is an independent community and each community has an independent label i.
(2) Next, we introduce a free control parameter β; the probability of the appearance of a new community is inversely proportional to
  $$P_{inverse}=\frac{n_{1}}{n+\beta }\times \frac{n_{2}}{n+\beta }\times \cdots \times \frac{n_{i}}{n+\beta }\times \frac{\beta }{n+\beta };$$ (1)
  here, $n_{i}$ is the number of nodes of community i and n is the number of nodes of the whole network.
(3) If the community is successfully formed, then we introduce a new node as the first member of the new community and randomly connect to m (m≤k) existing nodes from other communities. Hence, the label i of the new community is c+1, where c is the number of old communities. Otherwise, introduce a new node as a member of an old community. The community to be chosen is decided by its label number i; the bigger i means the later birth. In other words, the probability that a particular community is chosen is determined by its age, inversely proportional to $P_{inverse}$. The later born community is easy to be chosen with the bigger β then forming more communities.
(4) Next, the new node has a probability p to connect its own community nodes and 1−p to randomly connect to other community nodes. Here, p can be 1; due to the community, the first node has connected the node from other communities at step 2. If the number of nodes within a community is not enough to be connected by its new node, let the new node first connect all nodes within the community randomly and then connect the external nodes randomly.
(5) Repeat (2), (3), and (4) $n^{\prime }$ times and then the number of nodes is $n=k+n^{\prime }$.

Time is the key power in the BA model, the birth time determines the popularity of nodes, and the popularity of a node determines accretion of links. Hence, the richer get richer and the link resource distribution becomes more unequal. However, our model introduces the control parameter β to create new communities to get the resource and also controls both new and old communities to share the resource, in contrast to a mechanism in which high degree nodes absorb more links over time.

For a large network, only a small number of nodes may connect to other communities. However, a small number of nodes will struggle to make an impact on the community structure—or be strongly wired with their intended community. To accelerate the computation, we can delete these nodes and then add new nodes. Actually, in a real-world growing network, that some nodes only connect other communities is natural. Such as in the *SARS-Cov-2* haplotype evolutionary network,^25^ the haplotype h35 (group A) only connects other group B due to the relationship between them, and its community is uncovered. Hence, we can also ignore this phenomenon.

Furthermore, as depicted in Eq. (1), $P_{inverse}\to 0$ when β→∞, and the probability of the appearance of a new community tends to ∞ and the number of communities $N_{c}\to n$. On the contrary, $P_{inverse}\to \infty$ when β→0, and then the probability of the appearance of a new community tends to 0 and the number of communities $N_{c}\to k$. We set the probability of the appearance of a new community equal to 0 when β=0. Hence, β∈[0,∞). Here, we provide a fitting method for the relationship between the number of communities $N_{c}$ and the β,

$$N_{c}∼n^{\frac{\beta }{(k+\gamma )+\beta }};$$ (2)

**FIG. 1.**
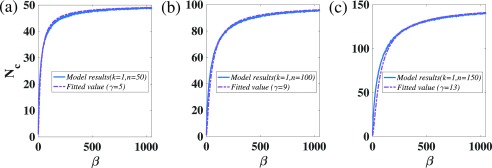
The relationship between the number of communities $N_{c}$ and the free control parameter β. The ordinate is the number of communities, and the abscissa is the free control parameter. The solid line is the result curve of our model, and the dashed line is the result of our fitting method. (a) We set m=1, p=0.9, k=1, n=50, and $\beta =\min(n_{i})$ for the model; actually, only k, n, and β are useful for our model curve. (b) We set m=1, p=0.9, k=1, n=100, and $\beta =\min(n_{i})$ for the model. (c) We set m=1, p=0.9, k=1, n=150, and $\beta =\min(n_{i})$ for the model.

γ is a free parameter for fitting. As shown in Fig. 1, we set k=1 and then change the number of nodes n. Results show that our fitting method can fit with the results of our model very well.

## III. FITNESS OF A GROWING MODEL

**FIG. 2.**
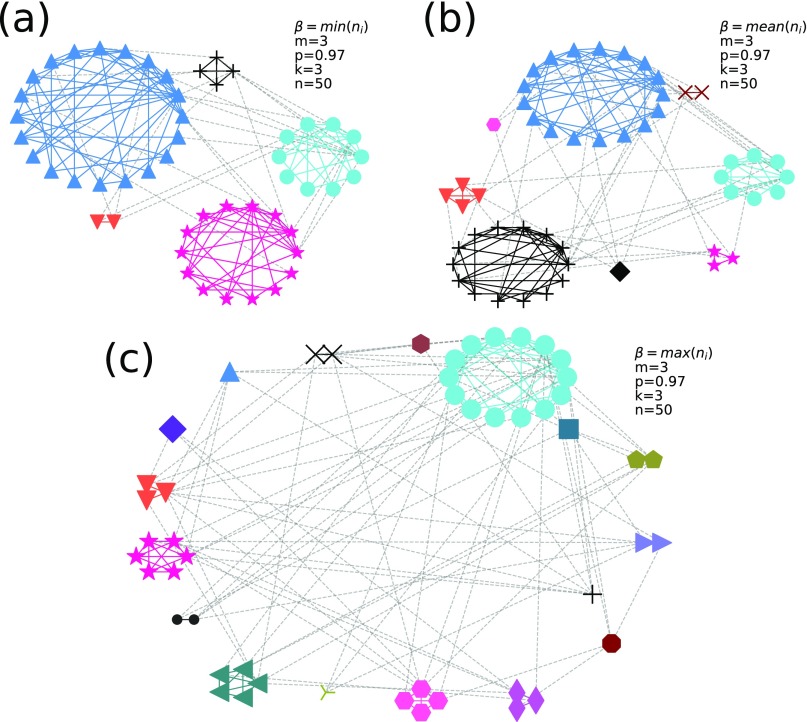
Network structures and the corresponding degree distributions for three regular networks of our model. In network structure figures, each community has its unique node shape, the dashed link is the link between communities, and the solid link is the link within the community. We set m=3, p=0.97, k=3, and n=50 for all networks and $\beta =\min(n_{i})$ for (a), $\beta =\text{\{mean\}}(n_{i})$ for (b), and $\beta =\max(n_{i})$ for (c). Here, a new node randomly connects the old node within the same community.

In this report, we give three examples (Fig. 2) to qualitatively interpret our network growth model. Here, we set the number of initial nodes k, the number of links per step (m), and the probability to connect to its own existing community (p) to be the same and only change the free control parameter β. We can see that with the increase in β, the number of communities increases, the size (number of nodes) of the biggest community decreases, and the degree of the largest hub node decreases (Table I). Furthermore, as shown in Table I, the increase in β sharply increases the number of communities and will greatly inhibit the growth of the community size. Hence, this will influence the degree of the hub node in each community. The node with the lowest degree still has the same degree value. This phenomenon states that the new nodes are more likely to get richer (more degrees), and more degree resources can be allocated to previously not richer nodes under our control.

**TABLE I.** The number of communities *N*_*c*_, the size of the biggest community *S*_max_, the highest degree *D*_max_, and the lowest degree *D*_min_ for each model network in Fig. 2.

	*N*_*c*_	*S*_max_	*D*_max_	*D*_min_
*β* = min(*n*_*i*_)	5	20	17	3
*β* = mean(*n*_*i*_)	8	17	14	3
*β* = max(*n*_*i*_)	17	14	13	3

**FIG. 3.**
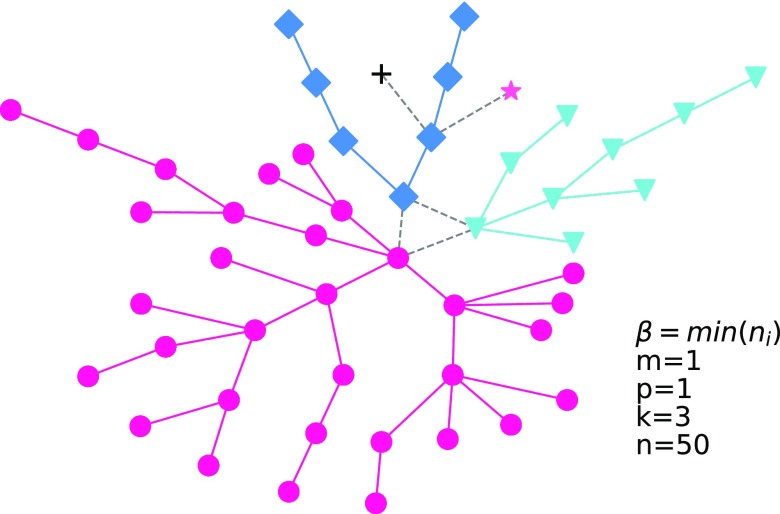
The network structures and the corresponding degree distribution for a tree-like network of our model. In the network structure figure, each community has its unique node shape, the dashed link is the link between communities, and the solid link is the link within the community. We set m=1, p=1, k=3, n=50, and $\beta =\min(n_{i})$ for it. Here, a new node randomly connects the old node within the same community.

Next, we set m=1 and p=1 and then directly construct a tree-like network.^16^ As shown in Fig. 3, the surprising result is that our model is very similar to a naturally evolving tree. Initial nodes are tree roots; with the time evolution, each branch is the community and the branch of the branch is the new community; and the rest can be deduced by analogy. The number of communities will increase with the increase in β; hence, we set $\beta =\text{\{min\}}(n_{i})$ to avoid a large number of communities.

### A. Regular networks

**FIG. 4.**
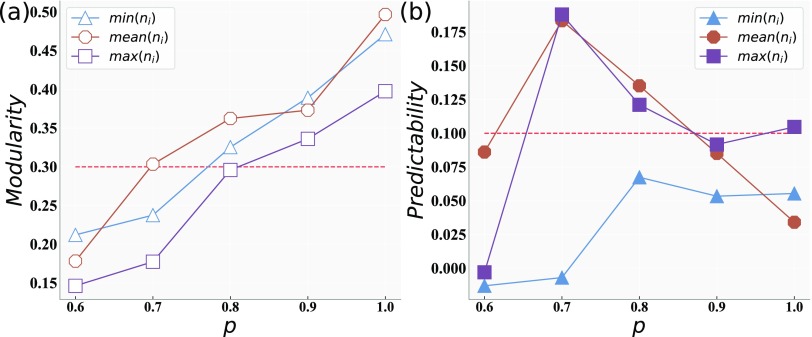
We set β as $\min(n_{i})$, $\text{\{mean\}}(n_{i})$, and $\max(n_{i})$, respectively. (a) is the Q value of different regular networks with the increase in p. The ordinate is the Q score of communities, and the abscissa is the p value. (b) is the Pr value of different regular networks with the increase in p. The ordinate is the Pr score of networks, and the abscissa is the p value.

To further discuss the fitness of our growing network model, we measure the community structure of our model by the default metric modularity.^23^ Here, we set k, m, and n as in Fig. 2. As shown in Fig. 4(a), we can see that the modularity value (Q) for all networks will increase with the increase in p. This means that, with the increase in p, our model will have a clearer so-called community structure. In detail, compared to $\beta =\min(n_{i})$ and $\beta =\text{\{mean\}}(n_{i})$, we can see that $\beta =\max(n_{i})$ has the worst performance under the metric of modularity. Moreover, $\beta =\text{\{mean\}}(n_{i})$ has the best performance under the metric of modularity, and the network has the clear community structure (Q is around 0.3^20^) if p>0.7. It should be noted that $\beta =\max(n_{i})$ also has a clear community structure when p>0.8.

Furthermore, many previous studies^26,27^ demonstrate that the community can improve the predictability of links. Hence, next, we measure the link predictability by the metric predictability (Pr^28^),

$$S_{pr}=\frac{\sum_{i=1}^{n}\frac{S_{in}^{i}-S_{all}^{i}}{S_{all}^{i}}}{n}.$$ (3)

Here, $S_{in}^{i}$ is the algorithm prediction accuracy of internal links between communities and $S_{all}^{i}$ is that of all links. We adopt the default metric AUC^29^ to measure our link prediction accuracy.^30^ In practice, it is not easy to improve the accuracy of a basis link prediction algorithm more than 10%; hence, the community has a very predictable structure if $S_{pr}>0.1$. In this report, we adopt three famous basic link prediction algorithms CN,^31^ LHN1,^32^ and HDI^15^ for regular networks.

As shown in Fig. 4(b), $\beta =\min(n_{i})$ has the worst performance under the metric of predictability. $\beta =\text{\{mean\}}(n_{i})$ has a better performance under the metric of predictability, and the community has the best predictability structure (Pr>0.1) if p is around the interval [0.7,0.8].

In summary, we suggest that setting $\beta =\text{\{mean\}}(n_{i})$ and 0.7≤p≤0.8 can construct a regular network with a clear community structure and a higher predictability of community links. However, we can also set $\beta =\max(n_{i})$ and p≈0.8 to further regulate the degree distribution. In addition, each real-world network has its own particular community structure; actually, not all networks have a high Q or Pr value. In other words, each network has its real and particular statistical characteristics. However, we can observe the statistical characteristic of the real network and then regulate β to construct a network with a similar Q value, a Pr value, and the number of communities to the real network.

### B. Tree-like network

#### 1. Growing model

**FIG. 5.**
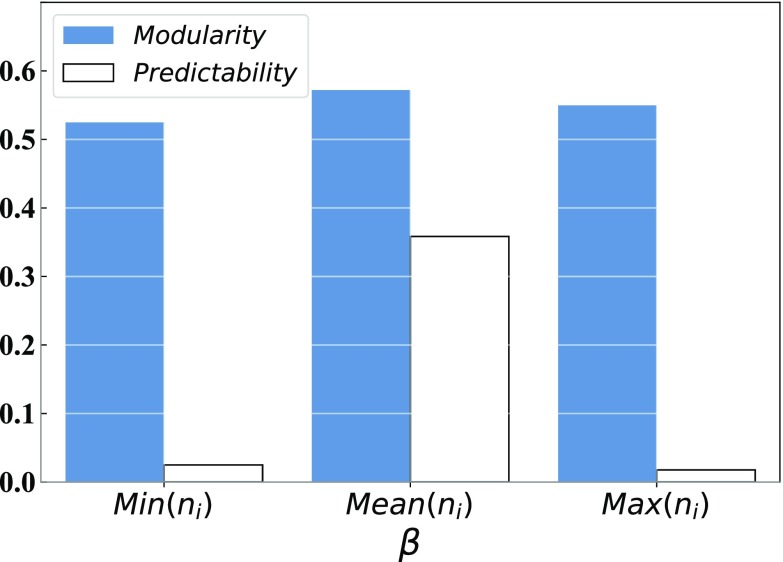
The Q value and the Pr value of three tree-like networks. We set β as $\min(n_{i})$, $\text{\{mean\}}(n_{i})$, and $\max(n_{i})$, respectively. The ordinate is the Q or Pr score of communities, and the abscissa is the β value.

Many real-world systems are naturally represented as tree-like networks with almost no closed triangular structure.^16^ Here, we set k, m, p, and n as shown in Fig. 3. We adopt the HEI (the algorithm for tree-like networks) as the basic link prediction algorithm.^16^ As depicted in Fig. 5, compared to $\beta =\min(n_{i})$ and $\beta =\text{\{mean\}}(n_{i})$, the performance of modularity and predictability both can achieve the best and high-quality (Q>0.3 and $S_{pr}>0.1$) performance when we setting $\beta =\text{\{mean\}}(n_{i})$. Hence, for tree-like networks, we suggest that set $\beta =\text{\{mean\}}(n_{i})$ can achieve a clear community structure and a higher predictability of community links.

#### 2. The haplotype evolutionary network of SARS-Cov-2

**FIG. 6.**
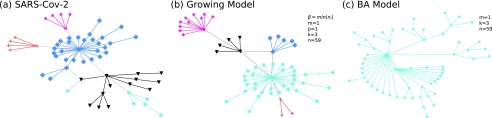
(a) is the network structure of *SARS-Cov-2* haplotypes. (b) is the network structure of our model, and we set m=1, p=1, k=3, n=59, and $\beta =\min(n_{i})$ for it. Here, a new node connects the oldest node within the same community with a 90% probability and randomly connects other old nodes within the same community. (c) is the network structure of the BA model, and we set m=1, k=3, and n=59.

Actually, the tree-like networks are ubiquitous in nature and human society.^16^ In particular, the virus haplotype evolutionary network is a natural tree-like network with new communities. In this report, we adopt the *SARS-Cov-2* haplotype evolutionary data^25^ as a real-world network, a node stands for one kind of a haplotype or a medium vector, and a link stands for an evolutionary relationship between two connected nodes. The *SARS-Cov-2* haplotype evolutionary network with 5 groups (communities) has 59 nodes (58 haplotypes and one medium vector mv2) and 59 links before 4 February 2020. Here, in the *SARS-Cov-2* network, we observe that the root node in each community is the hub node with the highest degrees; hence, we set that a new node connects the oldest node within the same community with a 90% probability. As depicted in Fig. 6, when we set $\beta =\min(n_{i})$ to avoid a large number of communities, n=59. Our model can construct a network that has the same number of communities, the lowest degree, and the maximum depth of the tree within a community as the real evolutionary network. Furthermore, as shown in Table II, our model network^33^ has the similar modularity value, the predictability value, and the highest degree as that of the real network. Results show that our model can fit well with the real-world evolving network that has new communities. In addition, we can also see that the Barabasi–Albert (BA)^11^ network cannot grow communities [Fig. 6(c)], and the highest degree value (18) is far less than that of the real network (30). Hence, we suggest that the BA model cannot fit with the real-world evolving network that has new communities.

**TABLE II.** The number of communities *N*_*c*_, the maximum depth of the tree within a community *L*_max_, the size of the biggest community *S*_max_, the highest degree *D*_max_, the lowest degree *D*_min_, the *modularity* value *Q*, and the *predictability* value *Pr* for networks in Fig. 6.

	*N*_*c*_	*L*_max_	*S*_max_	*D*_max_	*D*_min_	*Q*	*Pr*
SARS-Cov-2	5	2	30	27	1	0.5471	0.0082
Growing model	5	2	34	31	1	0.5208	0.0071

### C. Karate club network

**FIG. 7.**
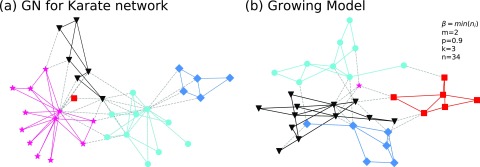
(a) is the network structure of the karate club. (b) is the network structure of our model, and we set m=2, p=0.9, k=3, n=34, and $\beta =\min(n_{i})$ for it. Here, a new node randomly connects the old node within the same community.

Dissimilar to the haplotype evolutionary network of SARS-Cov-2, previous famous traditional networks have no natural communities. However, researchers proposed many detection algorithms to uncover their defined communities. In this report, we adopt the karate club network^34^ as the real regular network: A node represents one club user, and a link indicates that there is a relationship between a pair of nodes. The karate club network is the common used data and has been widely applied for community detection.^17^ Moreover, we adopt the foundational community detection algorithm, the Girvan–Newman (GN) algorithm,^17^ to detect the karate club network. As depicted in Fig. 7, when we set $\beta =\min(n_{i})$ to avoid a large number of communities, k=3, m=2, and n=34. Our model can construct a network that has the same number of communities and the size of the biggest community as the real karate club network. Surprising, our model and the real network each have a community with only one node. Furthermore, as shown in Table III, our model network has a similar modularity value, a predictability value, and the lowest degree as that of the real network. Results show that our model can also fit with the traditional network, which has been widely used for community detection.

**TABLE III.** The number of communities *N*_*c*_, the size of the biggest community *S*_max_, the size of the smallest community *S*_min_, the highest degree *D*_max_, the lowest degree *D*_min_, the *modularity* value *Q*, and the *predictability* value *Pr* for networks in Fig. 7.

	*N*_*c*_	*S*_max_	*S*_min_	*D*_max_	*D*_min_	*Q*	*Pr*
Karate network	5	12	1	17	1	0.4013	0.0581
Growing model	5	12	1	11	2	0.4746	0.0554

## IV. CONCLUSION AND DISCUSSION

Growing network models or static network models with communities have been proposed many times, but a fitness growth model that incorporates the birth of communities and can achieve tree-like networks is very rare. Our model exhibits a clear community structure, the more predictable links of communities, and the varying degree distribution at the same time. Particularly, in the construction of tree-like networks, our growing model can grow the branches and leafs for the tree-like network, and the almost perfect new branches or leafs that are precisely the new communities. More importantly, compared to the *SARS-Cov-2* haplotype evolutionary network, our model can construct a network with the same number of communities and a similar network structure. In addition, our model also can fit with the common used network of community detection.

“Rich get richer” is the common sense for network science; however, new communities often expand rapidly in the real world. Our work helps us to utilize the new community phenomenon and to contribute a novel network model. Another possible way is that using the increase in new communities helps us improve the link prediction accuracy of tree-like networks.

## Data Availability

The genomic data used in this study are available from the article W.-B. Yu, G.-D. Tang, L. Zhang, and R. T. Corlett, “Decoding evolution and transmissions of novel pneumonia coronavirus using the whole genomic data,” ChinaXiv:202002.00033 (2020) and the associated supplementary material. The karate club data used in this study are available from the article M. Girvan and M. E. Newman, “Community structure in social and biological networks,” Proc. Natl. Acad. Sci. U.S.A. **99**, 7821–7826 (2002) and the associated supplementary material.
